# Transcription Factor EB (TFEB) Expression and Localization in the Third-Trimester Placenta †

**DOI:** 10.3390/ijms262110294

**Published:** 2025-10-22

**Authors:** Cinzia Giacometti, Alessandro Ambrosi, Serena Cavaliere, Anna Caliò, Daniele Mautone, Guido Martignoni

**Affiliations:** 1 Pathology Unit, Pederzoli Hospital, Peschiera del Garda, 37019 Verona, Italy; guido.martignoni@univr.it; 2 IRCCS San Raffaele Scientific Institute, Vita-Salute San Raffaele University, 20132 Milano, Italy; ambrosi.alessandro@hsr.it; 3 Gynecology & Obstetrics Unit, Pederzoli Hospital, Peschiera del Garda, 37019 Verona, Italy; serena.cavaliere@ospedalepederzoli.it (S.C.); daniele.mautone@ospedalepederzoli.it (D.M.); 4 Pathology Unit, Department of Diagnostic and Public Health, University of Verona, 37126 Verona, Italy; anna.calio@univr.it

**Keywords:** gestational diabetes, preeclampsia, TFEB, immunohistochemistry

## Abstract

Transcription factor EB (TFEB) is expressed at high levels in the trophoblast cells of the placenta, where it plays a critical role in regulating normal vascularization. Preeclampsia (PE) is a severe complication of pregnancy with a high incidence of maternal and fetal morbidity and mortality. Gestational diabetes (GD) is a metabolic disease that can affect placental villous maturation and villous vascularity. We analyzed the expression of three different antibodies: TFEB from Invitrogen (TFEB-INV), which detects endogenous levels of TFEB only when phosphorylated at Ser211; TFEB from Bethyl Labs (TFEB-B), which recognizes and binds E-box sequences; and TFEB from Santa Cruz (C-6) (TFEB-SC), which is specifically used for epitope mapping between 440 and 470. We evaluated the presence/absence of TFEB in six placental districts: syncytiotrophoblast (STB), cytotrophoblast (CTB), extravillous trophoblast (EVT), syncytial knots, stem villi vessels, and villous capillaries. TFEB-B was significantly expressed in the stem villi vessels, STB, and villi vessels of GD cases. The lack of TFEB expression in late-onset PE appears to corroborate the role of TFEB in vascular remodeling during placental development. The positive results in STB and vessels in GD cases, regardless of the histological diagnosis, may suggest that the expression of TFEB mitigates hypoxic injury via the Akt/mTOR pathway.

## 1. Introduction

Preeclampsia (PE) is a hypertensive pregnancy-related disorder characterized by a spectrum of effects that can cause maternal and neonatal morbidity and mortality; the only definite treatment for this disorder is delivery [1,2]. In early-onset PE (<34 weeks of gestation), the main factor is inadequate placentation, which causes reduced uteroplacental supply. In late-onset PE (≥34 weeks of gestation), placentation is believed to be normal but there is increased fetoplacental demand. In both cases, a utero-placenta mismatch occurs (i.e., demand exceeds supply). Based on these premises, the placenta releases stress-related factors from placental syncytiotrophoblasts (STBs), leading to an imbalance in circulating levels of proangiogenic placental growth factor and antiangiogenic soluble fms-like tyrosine kinase 1 (sFlt-1) [3,4]. sFTL-1 is the most well-known biomarker for PE [5,6], which is primarily produced by STBs in response to oxidative stress, endoplasmic reticulum (ER) stress, and hypoxia [7,8,9]. In recent years, emerging theories about the pathophysiology of PE have focused on the roles of syncytiotrophoblast stress [3,10,11] and autophagy [12]. Hypoxia represents physiological stress during early pregnancy, promoting invasion, vascular remodeling, and the activation of autophagy in primary extravillous trophoblast (EVT) cells, both in vitro and in vivo [13,14]. Conversely, autophagy-deficient EVT cells showed impaired invasion and vascular remodeling [14]. The histological hallmark of intrauterine growth restrictions (IUGR) and preeclampsia is maternal vascular malperfusion of the placental bed (MVM), characterized by both gross (placental hypoplasia, infarction, and retroplacental hemorrhage) and microscopic findings (abnormalities in villous development such as distal villous hypoplasia and accelerated villous maturation), as well as decidual vascular abnormalities (decidual arteriopathy) [15].

Gestational diabetes mellitus (GDM) is a condition characterized by glucose intolerance first diagnosed during pregnancy, leading to maternal hyperglycemia. This condition occurs in about 15% of pregnancies in both developed and developing countries [16]. Although GDM resolves after birth, this disorder is linked to changes during prenatal development, perinatal alterations (e.g., macrosomia, insulin resistance, and higher systolic blood pressure), and health conditions in adulthood (e.g., diabetes, obesity, dyslipidemia, hypertension, and metabolic syndrome) [17,18]. The histological hallmark of GDM’s impact on placental maturation is delayed villous maturation (DVM), which also includes the reduced formation of vasculo-syncytial membranes, the presence of multiple centrally located capillaries, and a variable extent of chorangiosis [19].

Transcription factor EB (TFEB) is one of the four members of the MiT family of transcription factors, which includes MITF1, TFE3, and TFEC-L. The protein structure of TFEB is conserved across all family members and consists of a basic helixloophelixleucine zipper dimerization motif (bHLH-zip), a transactivation domain, and a basic region involved in DNA binding, which recognizes E-box-type DNA sequences [20,21]. The role of the MiT family in regulating cellular processes has been extensively studied [20,22]. TFEB is crucial for regulating fundamental cellular processes, including lysosomal biogenesis and autophagy. The subcellular location and activity of this transcription factor are regulated by the mechanistic target of rapamycin (mTOR)-mediated phosphorylation, which occurs at the lysosomal surface [22]. Phosphorylated TFEB stays in the cytoplasm, while dephosphorylated TFEB moves to the nucleus to turn on target gene transcription. Cellular metabolism and energy levels are thus regulated through a TFEB lysosome-to-nucleus signaling pathway [12,13]. TFEB is known to be essential for normal placental vascularization. TFEB-knockout mice die at 10 days of intrauterine life, and the placentas of these knockout mice do not express vascular endothelial growth factor (VEGF), which is necessary for embryonic and extraembryonic vasculogenesis [23]. This result is supported by a recent paper from Doronzo et al. [24], which indicates that TFEB regulates the cell cycle/vascular endothelial growth factor receptor 2 (VEGFR2) pathway, thereby reducing endothelial proliferation.

Understanding the role of TFEB in early placentation could provide a molecular rationale for PE patients and identify a potential treatment [15]. However, this understanding mainly (or only) applies to early-onset PE, in which defective placentation is a well-known and recognizable mechanism of hypoxic injury and impaired placental development [25]. In this scenario, the role of TFEB and autophagy failure in the development of PE appears to be well-supported in the literature and in numerous experimental studies [12].

However, hypoxic injury is not solely related to defective placentation but also represents a well-recognized risk factor for fetal growth restriction in GD [26], obesity [27], and high-altitude pregnancies [28], as many different maternal risk factors (such as obesity, diabetes, and hypertension) may contribute to an elevated vascular inflammatory response and subsequent STB oxidative stress [29]. While many experimental studies on cell lines and isolated placental tissue have examined the role of TFEB in the early stages of placentation, its function in the progression, maintenance, and outcome of pregnancy remains largely unknown, as no study has documented its presence or localization in third-trimester placentas. To enhance our understanding of hypoxic mechanisms in developing pregnancies, this study aimed to examine the localization of TFEB in third-trimester placentas under two distinct and highly hypoxic conditions: late-onset preeclampsia and gestational diabetes. These two conditions differ not only clinically but also from a pathogenetic and molecular perspective, with distinct and recognized hypoxic pathways, despite the recent “two-stage” theory of preeclampsia, which acknowledges a common final step even when starting from different conditions: defective placentation or placental size problems.

This article is a revised and expanded version of an abstract entitled “Transcription Factor EB (TFEB) Expression and Localization in the Third-Trimester Preeclamptic and Diabetic Placenta,” which was presented at the 114th Annual Meeting of the United States and Canadian Academy of Pathology, held in Boston from the 24–27 March 2025 [25]. The revision of the previous project changed the population studied (with the exclusion of two possibly confounding cases), with the subsequent acquisition of some previously lacking clinical information, a simplified scoring system (to increase reproducibility), and a more extensive statistical analysis. The abstract is listed in the References for comparison.

## 2. Results

### 2.1. Patients’ Characteristics and Clinical Correlations

The patient, newborn, and delivery characteristics are summarized in Table 1.

Ten consecutive cases of late-onset PE and ten consecutive cases of GD were retrieved from the placentas submitted to the Pathology Laboratory between April and September, 2024, according to guidelines and hospital requirements [24]. Of these samples, two cases were excluded due to incomplete clinical or laboratory data, and one was excluded because of fragmentation during delivery, resulting from manual removal of the afterbirth. All remaining placentas (*n* = 17) fulfilled the inclusion/exclusion criteria: nine placentas with a clinical diagnosis of late-onset PE and eight placentas from diagnosed GD mothers.

The resulting sample size allowed a test power of 1−β= 0.95 with respect to Cohen’s effect size d= 1.45 and a significance level α= 0.05.

The histological diagnoses were MVM in 11 cases, DVM in 1 case, mixed features of DVM and MVM in 4 cases, and mixed features of MVM and FVM in 1 case.

We found evidence for an association between a clinical diagnosis of PE and its histological counterpart (MVM) (*p* = 0.0014). A clinical diagnosis of GD, on the other hand, was associated with different histological diagnoses: MVM in two cases, DVM in one case, mixed features of DVM and MVM in four cases, and mixed features of MVM and FVM in one case (Table A1).

### 2.2. TFEB Expression and Correlations

TFEB-INV was entirely negative in all cases and all compartments studied. This result suggests that the inactive form of TFEB is not detectable in third-trimester pathologic placentas.

**Figure 1:**
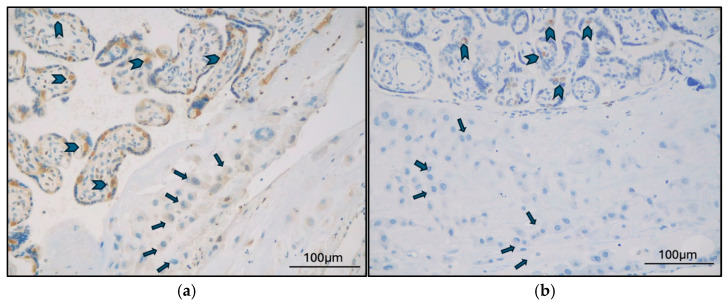
(**a**) Late-onset PE case (MVM), TFEB-B expression in CTB (arrowheads), and negative ETB (arrows) (original magnification 200×); (**b**) GD case (MVM+DVM), TFEB-SC expression in CTB (arrowheads), and negative EVT (arrows) (original magnification 200×).

**Figure 2:**
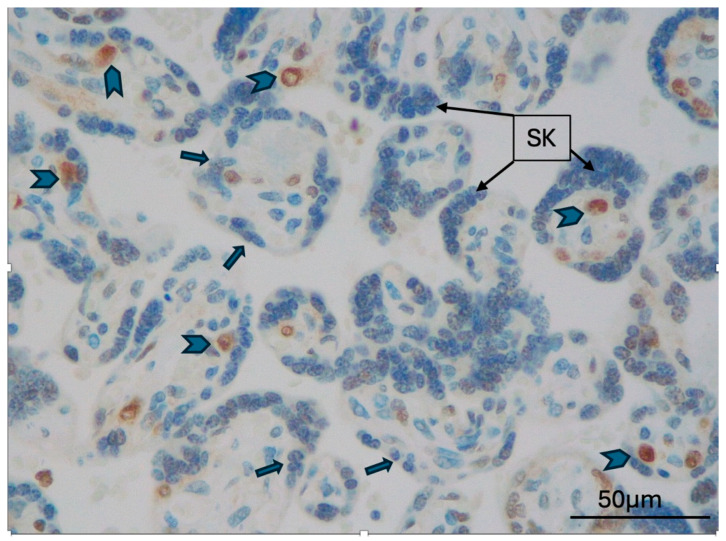
Late-onset PE case (MVM), TFEB-SC expression in CTB (arrowheads), negative STB (arrows), and negative syncytial knots (SK) (original magnification 400×).

The expression of TFEB-B and TFEB-SC was first analyzed according to localization in different placental districts (Table A2). TFEB-B and TFEB-SC were both consistently expressed in all cases in CTB; the expression of both antibodies was consistently absent in all EVT cases (Figure 1). Additionally, TFEB-SC was negative in syncytial knots in all cases (Figure 2), while TFEB-B was positive in two cases (2 GD, 0 PE; *p* = 0.205).

TFEB-B and TFEB-SC were expressed in STB only in three cases (3 GD; 0 PE) and two cases, respectively (0 GD, 2 PE) (*p* = 0.65).

**Figure 3:**
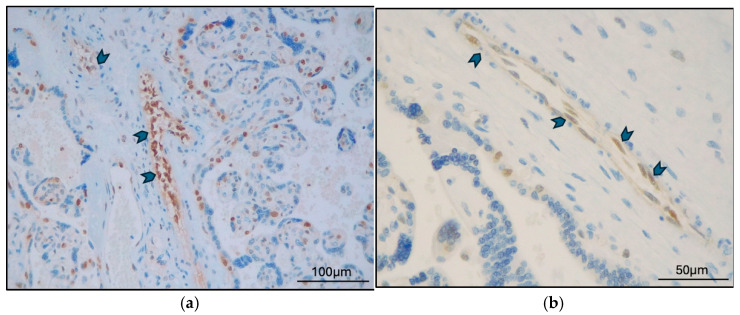
GD case (MVM+DVM), (**a**) TFEB-B expression in stem villi vessels (arrowheads) (Original magnification 400×) and (**b**) TFEB-SC expression in stem villi vessels (arrowheads) (Original magnification 200×).

TFEB-B (Figure 3a) was expressed in stem villi vessels in 10 of 17 cases (7 GD cases and 3 PE cases). In comparison, TFEB-SC (Figure 3b) was expressed in stem villi vessels in 2 of 17 cases (1 GD case and 1 PE case) (*p* = 0.002). In villi vessels, TFEB-B was expressed in five cases and TFEB-SC in one (*p* = 0.045).

No other statistical significance was found in the expression of TFEB-B across the different districts analyzed or in comparison with TFEB-SC. All the results and corresponding *p*-values are reported in Table A2.

We then analyzed the expression of TFEB according to the clinical diagnosis (late-onset PE and GD).

TFEB-SC expression was not statistically different in any compartment between GD and PE cases (Table A3).

TFEB-B was expressed in stem villi vessels in seven GD cases and three PE cases (*p* = 0.049). Expression in the other districts was not statistically significant (Table A3).

**Figure 4:**
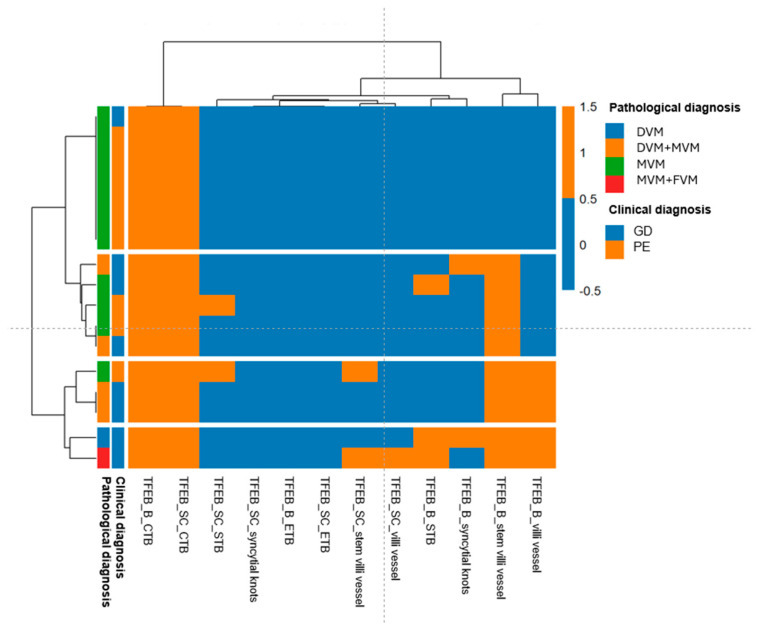
Heatmap of observed values (Hamming distance). Hamming distance was used to analyze the pairwise dissimilarities between patients and between placental districts. This analysis aimed to identify patterns of TFEB expression that were similar or different across clinical diagnosis, pathological diagnosis, and the analyzed placental districts.

Our analysis of TFEB expression in relation to histological diagnosis, clinical diagnosis, and placental regions revealed that TFEB-B was not present in the vascular compartment of MVM cases, which aligned with a PE clinical diagnosis. On the other hand, TFEB-B was statistically significantly expressed in the stem villi vessels (*p* = 0.04) and villi vessels (*p* = 0.02) of GD cases, irrespective of the histological diagnosis (Figure 4; Supplementary Materials).

The complete data set is available in the Supplementary Materials.

## 3. Discussion

When considering placental pathology, it should be noted that the placenta is a unique and “transient” organ. The placenta is a fetal organ that develops inside the maternal environment (the uterus). During development, the feto-placental unit undergoes significant endocrine and metabolic activity, which interplays with and conditions maternal immune and metabolic adaptive responses. Additionally, the maternal environment plays a crucial role in conditioning fetal well-being, development, and growth. In the nine months of intrauterine life, the fetus and the placenta undergo dramatic changes, from a complex composed of a few cells to a complete human being.

Many different molecular pathways, molecules, and hormones and an extraordinarily complex and highly controlled mechanism are involved during implantation to guarantee correct blood and oxygen supply to the developing embryo. One of the key players in this process is TFEB.

The essential role of TFEB in placental development has remained unclear for more than 20 years after the first intriguing observation suggested by Steingrimsson et al., who showed that homozygous TFEB knock-out mice had defects in the ability of their embryonic vasculature to invade the placenta, indicating that problems with oxygen and nutrient exchange could cause the observed lethality [23].

More recently, TFEB has also been identified as a regulator of placental development and function by coordinating both the transcriptional program involved in trophoblast fusion and the development of endocrine function, which are essential for the energy needs of embryonic growth [30].

However, despite advances in understanding the role of TFEB in human placentation, little is known about its role in the phases after placentation.

In the recent two-stage model of PE, a different etiology for late-onset PE has been proposed. While early-onset PE is thought to be strictly linked to poor placentation, late-onset PE appears to occur when the placenta exceeds uterine capacity, the terminal villi are compressed, and intervillous perfusion is impaired [31]. According to the two-stage theory, preeclampsia should be seen as a disorder related to STB stress, not just as a placental disorder. STB stress might be caused by maternal malperfusion due to defective placentation in early pregnancy (as in early-onset PE) or by late pregnancy factors related to placental growth and compression, which lead to malperfusion and hypoxia (as in late-onset PE) [10,11,31]. This theory is fundamental to understanding that late-onset PE has a highly complex pathology, including many different pre-gestational and pregnancy-related risk factors, and differentiates early-onset from late-onset PE [31].

Based on the two-stage theory, we hypothesized that STB oxidative stress could be examined in late-onset PE and GD cases, based on investigating potential responses to chronic hypoxia via TFEB. The rationale for this choice was based on the fact that placentas from these two different clinical scenarios (PE and GD) can also exhibit unique histological changes, even if it is well known that the histological hallmark of PE (i.e., MVM) can often be found in normal pregnancies, cases of GD, or placentas from hypertensive mothers. Similarly, DVM, the histological hallmark of GD, can be found in non-diabetic mothers with excessive weight gain during pregnancy, twin gestations, or intermittent cord obstruction.

We analyzed the expression of TFEB in various placental districts involved in placentation and vascularization, including STB, CTB, EVT, and the vessels of stem villi and terminal villi.

When examining the histological diagnoses, all PE cases were classified as MVM according to Amsterdam’s classification. In contrast, GD cases were classified as either DVM (in only one case), MVM, or a combination of both, indicating that GD is a more diverse entity.

Our in vivo analyses revealed that TFEB was consistently absent in STB and vascular in late-onset PE cases. The absence of TFEB in STB may indicate the placental tissue’s inability to respond to chronic hypoxic injury. This factor could also be inferred from the histological diagnoses, in which the MVM pattern was predominant. One of the key features of MVM is the presence of pen-like, slender villi (so-called distal villous hypoplasia), along with an increase in syncytial knots [19]. The MVM pattern is usually linked to preeclampsia, fetal growth restriction, and hypertensive disorders—all conditions that are vulnerable to decreased placental blood and oxygen supply. Syncytial knots are stress-response markers [32] that can be induced in vitro by hypoxia and oxygen species [33] but are transcriptionally inactive aggregates of nuclei. Based on these observations, it is not surprising that syncytial knots were consistently negative for TFEB in all cases (late-onset PE and GD) when tested with the three different antibodies.

So far, the lack of TFEB in STB seems consistent with the idea that a defective TFEB could be involved in developing PE, regardless of whether it occurs (early-onset or late-onset).

The other striking result is the expression of TFEB in STB and the vascular compartment analyzed (stem villi vessels and the villi vessels) of the GD placentas.

The involvement of TFEB in regulating vascular development was demonstrated in endothelial cells through deregulation of the VEGFA–VEGFR pathway in the placental cells of TFEB-knockout mice [24] and in GD placentas [34]. In our samples, GD cases showed localization of TFEB in the endothelium of stem villi vessels and villi vessels. This localization differed from that in late-onset PE cases and occurred irrespective of the histological diagnosis. GD cases nearly always showed some degree of DVM, whose hallmark is the presence of large intermediate-like villi with centrally located capillaries, sometimes accompanied by a striking increase in neoangiogenesis within the villi themselves (chorangiosis). Compelling evidence for the pro-angiogenic effect of TFEB has emerged in recent years. TFEB has been recognized as a positive regulator of angiogenesis through activation of AMP-activated protein kinase [35]. In a diabetic placenta, the binding of insulin to insulin-receptor B decreases the activity of mTOR [36]. However, insulin resistance is not the only harmful factor impacting the fetoplacental endothelium in GD placentas. Dysfunction of the endothelium is a well-recognized key feature of GD placentas [37]. Hypothetically, in GD placentas, the decrease in mTOR caused by insulin resistance may lead to dephosphorylation of TFEB and its translocation to the nucleus [21]. We believe that this mechanism, which likely differs in late-onset PE placentas, may explain both the increased vascularization of GD placentas and the reduced extent of hypoxic damage, even though neither dietary restriction nor insulin therapy, despite restoring euglycemia, can prevent or resolve endothelial damage [38]. The histological findings might be similar in the two populations studied. Still, the immunohistochemical findings relate to the clinical diagnoses rather than the histological diagnoses, highlighting the significant difference between a late-onset PE and a GD patient. We selected a relatively clear-cut population, avoiding the inclusion of other confounding or different factors. In this context, we hypothesize that the hypoxic injury, present in both groups, is likely mitigated by the activation of TFEB in GD placentas. We believe that a deeper understanding of the mTOR pathway in GD cases is necessary, especially regarding the immunophenotypic profile of more complex cases, which often involve a GD patient with hypertension or late-onset PE.

## 4. Materials and Methods

This retrospective observational study did not entail any changes in therapeutic or diagnostic procedures and was conducted in accordance with the Declaration of Helsinki. Only intact placentas and patients with available clinical data and analyses were considered in this study. Placentas were collected from patients with a diagnosis of (1) late onset preeclampsia, diagnosed according to the relevant guidelines [39]; (2) GD, in a two-hour test with a 75 g syrup glucose solution, was defined by the presence of at least one glycemic level above normal values equal to or higher than 92 mg/dL (5.11 mmol/L) immediately after (time 0) and/or equal to or higher than 180 mg/dL (10 mmol/L) after 60 min and/or equal to or higher than 153 mg/dL (8.5 mmol/L) after 120 min. All patients had no other co-morbidities. The placentas were collected after delivery at the Pederzoli Hospital (Peschiera del Garda, Verona, Italy). All the delivered placentas were formalin-fixed and paraffin-embedded after 2–4 days of 4% buffered formalin fixation. Placental sampling was conducted according to the Amsterdam protocol [15], which was modified as follows. A total of six samples were collected from each placenta: one sample of membranes (membrane roll) and the umbilical cord (proximal, intermediate, and distal, near the cord insertion), one sample including cord insertion, three samples of placental parenchyma (two center–parenchymal, representing one of the most and least normal areas), and one para-central sample. All diagnoses were rendered according to the Amsterdam protocol [15] as follows: delayed villous maturation (DVM), maternal vascular malperfusion (MVM), fetal vascular malperfusion, or a combination of two (or more) diagnoses (e.g., MVM + DVM, MVM + FVM).

To study the expression and localization of TFEB in the human placenta, we used three different antibodies: TFEB from Invitrogen–Thermo Fisher (Waltham, MA, USA) (TFEB-INV) (rabbit/IgG, polyclonal, 1 mg/mL, dilution 1:100), which detects endogenous TFEB levels only when phosphorylated at Ser211, indicating its inactive status (phosphorylated, cytoplasmic); TFEB from Bethyl Laboratories (Montgomery, TX, USA) (TFEB-B) (rabbit/IgG, polyclonal, 1 mg/mL), which specifically recognizes and binds to E-box sequences (3′-CANNTG-5′); and TFEB from Santa Cruz Biotechnology (Dallas, TX, USA) (C-6) (TFEB-SC), which targets an epitope between amino acids 440 and 470 within an internal region near the -COOH terminal of human TFEB (mouse monoclonal, clone sc-166736, 200 μg/mL, dilution 1:100). Immunohistochemistry was performed following the manufacturer’s instructions, using a citrate buffer (pH 6.0) for epitope retrieval, as recommended for FFPE tissue sections. Incubation without a primary antibody served as negative control. All three antibodies are intended for research use only. The image acquisition was conducted by Microscope Leica ICC50W (Leica, Wetzlar, Germany) and software Leica Suite V4.13.

In the mid portion of a chosen slide of placental parenchyma, we assessed the presence/absence of TFEB in five different placental districts: villous STB, villous cytotrophoblast cells (CTB), EVT, and endothelial cells of stem villi vessels and villous vessels. The adopted score was dichotomic (absence = 0; presence = 1).

Continuous variables are summarized as the median and interquartile range (IQR; 1st–3rd quartile), while categorical variables are summarized as frequencies and percentages. Between-group differences were assessed using a Mann–Whitney test for continuous outcomes χ^2^, McNemar or Fisher’s exact test for categoric variables, as opportune. All *p*-values were computed via permutation procedures to avoid any distributional assumptions or asymptotic approximation; *p*-values < 0.05 were considered significant.

Pairwise dissimilarities between patients and between placental districts were quantified using Hamming distance. To further investigate the presence of homogeneous groups, unsupervised hierarchical clustering was applied, and the results were visualized as a heatmap with associated dendrograms.

All analyses and graphics were performed using R (version 4.5.1, Library Coin).

## 5. Conclusions

Characterizing potential signaling mechanisms in GD and late-onset PE may help to elucidate complex pathologies that are not yet understood.

In our study, TFEB-B expression was significantly higher in GD cases across all histological diagnoses, in both STB and vascular compartments. However, when analyzed via histological diagnosis, TFEB-B expression was absent in the vascular compartment of MVM cases, which aligned with the clinical diagnosis of PE. Our work, which focuses on placental tissue rather than cultured cells, supports the hypothesis that the absence of TFEB expression is related to maladaptive placental development, leading to decreased vascular density in third-trimester placentas. The expression of TFEB in endothelial cells in GD placentas may indicate that these cells play a pivotal role in microvascular adaptive mechanisms. Since the placental microenvironment is highly complex, many pathways and metabolic mechanisms likely depend on changes observed at both cellular and phenotypic levels in GDM. We previously described a “proliferative”/antiapoptotic pattern (high Ki-67, PHH3, and p57 counts) in GD placentas compared to those in non-GD cases [40]. A combination of the unique GD immunophenotype (hENT1 negative, TFEB positive) could strongly support the identification of GD placentas, regardless of morphological features and clinical history. Future studies should explore the expression of TFEB in non-clinically evident GD, early-onset PE and, possibly, normal placentas.

The lack of previous research on this topic further limited this study’s scope. Moreover, prior research relevant to our thesis is scarce, largely experimental, and based on cell cultures isolated from placental tissue. For this reason, we developed a novel approach utilizing placental tissue and immunohistochemistry.

The empirical results presented here should, however, be considered in light of certain limitations.

The retrospective nature of this study did not allow for the collection of precise and comprehensive potential data about the mother’s health and pregnancy. This problem could be easily addressed by prospectively enrolling patients and using appropriate surveys to explore dietary and lifestyle habits that may impact pregnancy outcomes. We also acknowledge that the sample size is limited due to resource constraints and the absence of a control group, which also turn into a lack of power in statistical tests. The lack of a control group was due to adherence to guidelines regarding placental referrals to the Pathology Laboratory [41]. Consequently, placentas from normal pregnancies were not available.

## Figures and Tables

**Table 1:** Patients, newborns, and placental characteristics.

Mothers and Deliveries	Number/Total (%)	Mean ± SD	Range
Age (years)		34.76 ± 5.09	29–45
Clinical diagnosis			
Late-onset PE	9/17 (53%)		
GD	8/17 (47%)		
Histological diagnosis			
MVM	11/17 (65%)		
DVM	1/17 (6%)		
MVM + DVM	4/17 (23%)		
MVM + FVM	1/17 (6%)		
Type of delivery			
Caesarian	15/17 (88%)		
Vaginal, induced/operative	1/17 (6%)		
Vaginal, spontaneous	1/17 (6%)		
**Newborns and Placentas**		**Mean ± SD**	**Range**
Gestational age (weeks ± days)		37.00 ± 14.70	31.57–40
Birth weight (grams)		2703.23 ± 550.81	1500–3650
APGAR score (1 min)		8.76 ± 1.30	6–10
APGAR score (5 min)		9.41 ± 0.93	7–10
Umbilical cord (arterial) pH		7.27 ± 0.09	7.07–7.4
Base excess (arterial)		−2.24 ± 4.32	−10.6–4.8
Placental weight (grams)		364.11 ± 82.58	210–470
Placental Weight Ratio		7.57 ± 1.40	5.62–10.93

## Data Availability

The raw data supporting the conclusions of this article are available in Supplementary Materials.

## Supplementary Materials

The following supporting information can be downloaded at: https://www.mdpi.com/article/10.3390/ijms262110294/s1.

## Abbreviations

The following abbreviations are used in this manuscript:

MVM	Maternal vascular malperfusion
DVM	Delayed villous maturation
FVM	Fetal vascular malperfusion
PE	Preeclampsia
GD	Gestational Diabetes
STB	Syncytiotrophoblast
CTB	Cytotrophoblast

## Appendix A

**Table A1:** Correlation between clinical and histological diagnosis (χ^2^ test).

	Histological Diagnosis (N. of Cases)					
Clinical Diagnosis	MVM	DVM	MVM + DVM	MVM + FVM	Total	*p* Value
Late-onset preeclampsia (N. of cases)	9	0	0	0	9	0.0014
Gestational diabetes (N. of cases)	2	1	4	1	8	
Total	11	1	4	1	17	

**Table A2:** Correlation between TFEB expression and localization (McNemar test).

**TFEB Type**	**Placental Districts (N. of TFEB Positive Expression/Total)**					
	**STB**	**CTB**	**EVT**	**Syncytial Knots**	**Stem Villi Vessel**	**Villi Vessels**
TFEB-B	3/17	17/17	0/17	2/17	10/17	5/17
TFEB-SC	2/17	17/17	0/17	0/17	2/17	1/17
*p* value	0.65	1	1	0.205	0.002	0.045

**Table A3:** Correlation between TFEB-B and TFEB-SC expression in different placental districts and clinical diagnosis (Exact Fisher’s Test).

		Clinical Diagnosis (N. of Cases/Total)		
TFEB Type	Placental District	DG	Late-Onset PE	*p* Value
TFEB-B	STB	3/8	0/9	0.08
	Syncytial knots	2/8	0/9	0.20
	Stem villi vessels	7/8	3/9	0.049
	Villi vessels	4/8	1/9	0.13
TFEB-SC	STB	0/8	2/9	0.47
	Syncytial knots	0/8	0/9	-
	Stem villi vessels	1/8	1/9	1
	Villi vessels	1/8	0/9	0.47
